# Desflurane impairs hippocampal learning on day 1 of exposure: a prospective laboratory study in rats

**DOI:** 10.1186/s12871-019-0793-8

**Published:** 2019-07-04

**Authors:** Ayako Tojo, Kazuhiro Uchimoto, Gaku Inagawa, Takahisa Goto

**Affiliations:** 1 Department of Anesthesiology and Critical Care Medicine, Yokohama City University Graduate School of Medicine, 3-9, Fukuura, Kanazawa-ku, Yokohama, Japan; 2 Department of Intensive Care, Yokohama City University Medical Centre, 4-57, Urafune-cho, Minami-ku, Yokohama, Japan; 3 Department of Anesthesiology, Yokohama Municipal Citizen’s Hospital, 56, Okazawa-cho, Hodogaya-ku, Yokohama, Japan

**Keywords:** Desflurane, Contextual learning, Hippocampal LTP, Synaptic GluR1

## Abstract

**Background:**

Quick and complete recovery of cognitive function after general anesthesia is desirable, particularly for working-age patients. Desflurane is less likely to have long-term effects than older-generation inhalational anesthetics, however, its short-term effects have not been fully investigated. Our objective was to elucidate the short-term effects of desflurane exposure on learning and memory in young adult rats.

**Methods:**

Seven-week old male Sprague–Dawley rats were exposed to air (control), or desflurane at 0.7 or 1.2 minimum alveolar concentration (MAC) for 2 h (day 0). The inhibitory avoidance (IA) test was performed on day 1 to delineate the effects on contextual learning. Separate groups of control and 1.2 MAC desflurane animals underwent the IA test on days 3 and 7 to examine the time-dependent changes. Because the IA test is known to be dependent on the long-term potentiation (LTP) of the hippocampus and the trafficking of the GluR1 subunit of the α-amino-3-hydroxy-5-methyl-4-isoxazolepropionic acid receptor into the synapses, the effects of 1.2 MAC desflurane on these phenomena were evaluated on day 1.

**Results:**

Desflurane at 1.2 MAC, but not 0.7 MAC, significantly decreased the IA latencies on day 1 compared with the control (one-way ANOVA, *F* [2,48] = 5.974, *P* = 0.005, post hoc Tukey’s, mean difference [95% confidence interval], control vs. 1.2 MAC, 168 [49.9 to 287], *P* = 0.004; control vs. 0.7 MAC, 67.5 [− 51.2 to 186], *P* = 0.362). The latencies were not affected on days 3 and 7 (day 3, control vs. desflurane, *P* = 0.861; day 7, control vs. desflurane, *P* > 0.999). Consistently, hippocampal LTP on day 1 was significantly suppressed in the desflurane group compared with the control group (*P* = 0.006). Moreover, immunoblotting analysis of synaptic GluR1 expression revealed that desflurane exposure significantly suppressed GluR1 delivery to the synapses after IA training.

**Conclusion:**

Exposure to a relatively high concentration of desflurane caused reversible learning and memory impairment in young adult rats associated with suppression of GluR1 delivery to the synapses in the hippocampus.

## Background

Postoperative cognitive dysfunction (POCD) occurs in not only aged adults but adults of all ages [1], and is known to decrease the levels of daily activity [2], increase mortality, and the risk of leaving the labor market [3]. Although the underlying mechanisms have not been completely elucidated, there is emerging evidence from both clinical trials [4–9] and experimental animal studies [10–16] suggesting the contribution of inhalational anesthetics to POCD.

We previously reported that isoflurane impairs hippocampal learning 7 days after exposure in young adult rats [15]. In contrast to isoflurane, desflurane is reported to induce neither neurotoxicity [16–21] nor clinical cognitive dysfunction [4], and is less likely to induce long-term effects on learning and memory in rats [14]. However, regarding cognitive function, these studies investigated the effects 48 h [4] or 1 week or longer [14] after the anesthetic exposure and the shorter-term effects have not been fully studied. Because rapid and complete recovery of cognitive function is highly desirable especially in working-age patients and in ambulatory settings, we considered it meaningful to evaluate the shorter-term effects of desflurane.

The aims of the present study were two-fold. First, we sought to elucidate the short-term (no longer than 7-day) effects of desflurane on learning and memory in young adult rats, by using the inhibitory avoidance (IA) test, a widely used and robust behavioral test of contextual learning. Second, because the IA test is known to be dependent on hippocampal long-term potentiation (LTP), and the synaptic increment of the GluR1 subunit of α-amino-3-hydroxy-5-methyl-4-isoxazolepropionic acid receptors (AMPAR) in the hippocampus [22, 23], we sought to elucidate the effects of desflurane on these phenomena. We previously demonstrated that isoflurane impairs IA learning and LTP, and modulates synaptic GluR1 7 days after the exposure [15]. Our hypothesis was that desflurane would have similar effects, which would occur earlier.

## Methods

### Subjects

All experiments were conducted with approval from the Institutional Animal Care and Use Committee of the Animal Research Center at the Yokohama City University Graduate School of Medicine, Yokohama, Japan (approval number: F-A-14-038, F-A-17-079). Care and experiments of animals were performed in accordance with the Guidelines for Proper Conduct of Animal Experiments (2006, Science Council of Japan), and the study adhered to the Animal Research: Reporting In Vivo Experiments guidelines.

Male Sprague–Dawley rats (Japan-SLC, Shizuoka, Japan), aged 7 weeks old, weighing 200–270 g (total *n* = 170) were used in the study. Rats were housed in a temperature- and humidity-controlled room in a 12-h light-dark cycle, with ad libitum water and food. All rats were purchased at least 1 week before they were used.

### Anesthesia

On day 0, rats were randomly allocated to the control or desflurane-exposure group for each experiment. Anesthesia was induced and maintained for 2 h in a translucent plastic chamber (length, 30 cm; width, 43 cm; height, 14 cm) with 9.6% or 5.6% desflurane, which corresponds to 1.2 or 0.7 minimum alveolar concentration (MAC) [14, 24], respectively, continuously flushed with a carrier gas consisting of oxygen and nitrogen (F_I_O_2_ = 0.40–0.45). Rats were allowed to breathe spontaneously; however CO_2_ level in the container was maintained at less than 3 mmHg. The fraction of inspiratory and end tidal gas concentration was monitored continuously with Capnomac ULTIMA monitor (Datex, Helsinki, Finland).

Heart rate (HR) and oxygen saturation (SaO_2_) were measured continuously during anesthesia with a Mouse OX™ Pulse Oximeter (Harvard Apparatus, Holliston, MA, USA). Rectal temperature was maintained at 37 ± 1 °C using a thermostatic bath (36 ± 1 °C).

As for the control group, rats were placed in the plastic chamber and flushed with the same carrier gas for 1 min. They were only exposed for 1 min, with the intention of exposing them to the same stress as that experienced by the desflurane-exposure rats before being anaesthetized.

### Physiological variables

Rats (*n* = 5) of separate groups were anesthetized with 1.2 MAC desflurane for 2 h under the same conditions as described in the previous paragraph, with a catheter inserted into the carotid artery to continuously monitor blood pressure. At the end, blood samples were collected in heparinized syringes and were immediately analyzed using the ABL800 FLEX blood gas analyzer (Radiometer, Tokyo, Japan) to confirm the values of pH, PaCO_2_, PaO_2_, and HCO_3_^−^. These rats were not used in any further studies.

### IA test

The IA test, a widely used behavioral task to evaluate hippocampus-dependent contextual learning, was first performed to delineate the effect of exposure to desflurane on day 1. Rats were randomly allocated to the control, 0.7 MAC, or 1.2 MAC desflurane-exposure group (*n* = 17 each), considering the concentration dependency. An identical apparatus (length, 27 cm; width, 45 cm; and height, 25 cm) to that used in the previous study [15], where lighted and dark shock boxes are separated by a trap door and placed in a sound-shielded room, was used. Next, the test was also performed on days 3 and 7 with the control or 1.2 MAC desflurane-exposure group (*n* = 16 and *n* = 15 each, respectively) to examine time-dependent changes.

**Fig. 1:**
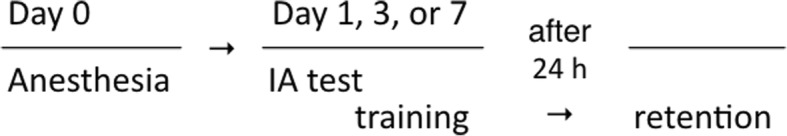
Experimental timeline of IA test

On day 1, the training session was performed. Each rat was initially placed in the lighted box and allowed to explore for 30 s. The trap door was silently opened, and soon after the rat went into the dark box, the door was closed. Subsequently, a scrambled electrical foot shock (1 s, 0.4 mA) was applied via the electrified floor using an SG-1000 shock generator (Melquest, Toyama, Japan). Fifteen seconds later, the rat was returned to its original cage. On day 2, the retention trial was performed. Each rat was again placed in the lighted box, and its latency to enter the dark box with all four paws was measured (maximum cut-off latency of 360 s). Longer retention test latencies are interpreted as better memory. The experimental time line is shown in Fig. 1.

All experiments were performed during the light phase (09:00–14:00) and were recorded with a video camera. The experimenter was blinded to the rat’s allocation group. Each rat was tested only once and euthanized with isoflurane and intraperitoneal pentobarbital after the test.

### Electrophysiological recordings

Separate groups of rats were anesthetized with 1.2 MAC desflurane (*n* = 7) or were handled as controls (*n* = 5) for brain slice preparation. Prior to decapitation, rats were briefly anesthetized with isoflurane for ethical reason, although brief anesthesia for surgical preparations or euthanasia has been reported to influence brain [25, 26]. Rat brains were rapidly removed and cut out to 300 μm coronal slices containing the dorsal hippocampus (Leika VT1000S; Leika Microsystems, Tokyo, Japan) in ice-cold dissection buffer. Subsequently, slices were transferred to artificial cerebrospinal fluid (ACSF; 22–25 °C), and were incubated at room temperature for 120 min. The dissection buffer and ACSF were bubbled (95% O_2_, 5% CO_2_) throughout the procedure.

The prepared brain slice was placed on an 8 × 8 array of planar microelectrodes (MED-P515A, Alpha MED Scientific, Osaka, Japan). Extracellular field excitatory postsynaptic potentials (fEPSPs) of the CA1 area, which were evoked by stimulating Schaffer collaterals, were acquired using the multi-electrode MED64 system (Alpha MED Scientific, Osaka, Japan). After 30 min of test stimulation (half maximal shock, every 60 s) to assess baseline stability, LTP was induced by applying high-frequency stimulation (100 Hz, 1 s), followed by test stimulation for 40 min. The LTP was evaluated by the mean slope of fEPSPs at 36–40 min from the high-frequency stimulation. Slices were continuously perfused with bubbled (95% O_2_, 5% CO_2_) ACSF throughout the experiment. Each slice was used only once. Although the experimenter was aware of the rats’ group allocation when performing the recordings, the data analyst was blinded.

### Quantitative immunoblotting

Separate groups of rats were anesthetized with 1.2 MAC desflurane (*n* = 20) or were handled as controls (*n* = 20) for immunoblot analysis of the AMPAR GluR1 subunit. Half rats of each group were IA-trained and the other half of each group were placed in a sound-shielded room but not IA-trained. Thirty minutes after the IA training or control condition (untrained), hippocampal tissue samples were promptly dissected using ice-cold dissection buffer.

Synaptoneurosomes, which are fractions enriched in synaptic elements, were prepared as previously described [22, 23, 27] . Immunoblotting was performed using primary antibodies against GluR1 (1:1000, Millipore, Temecula, CA, USA) and GAPDH (1:5000, Sigma-Aldrich, St Louis, MO, USA). All immunoblots and densitometry were performed with the experimenter blinded to the groups.

### Statistical analyses

The primary outcome was the IA latencies on day 1. The sample size of 17 per group was calculated for the three groups to be compared with a type 1 error of 0.05 and a power of 80% to detect a mean difference of 170 s, assuming the standard deviation to be 150 based on a pilot study where naive rats were IA trained. The normality of distribution was assessed with the Shapiro–Wilk test. For the primary outcome, the data were analyzed using one-way factorial analysis of variance (ANOVA) using desflurane concentration as the one factor followed by post hoc Tukey’s multiple comparison tests. To validate the time-dependent changes, the IA data were analyzed using two-way ANOVA using group and time point (day) as the two factors followed by post hoc Sidak’s multiple comparison tests. The data of the electrophysiological recordings were analyzed using t test. The immunoblot data were analyzed using two-way factorial ANOVA using group and IA training as the two factors followed by post hoc Sidak’s multiple comparison tests. All data are reported as mean ± standard error of mean (SEM). All statistical analyses were performed using the GraphPad PRISM version 6.0 (La Jolla, CA, USA), and *P* < 0.05 was considered statistically significant.

## Results

### Physiological variables

**Fig. 2:**
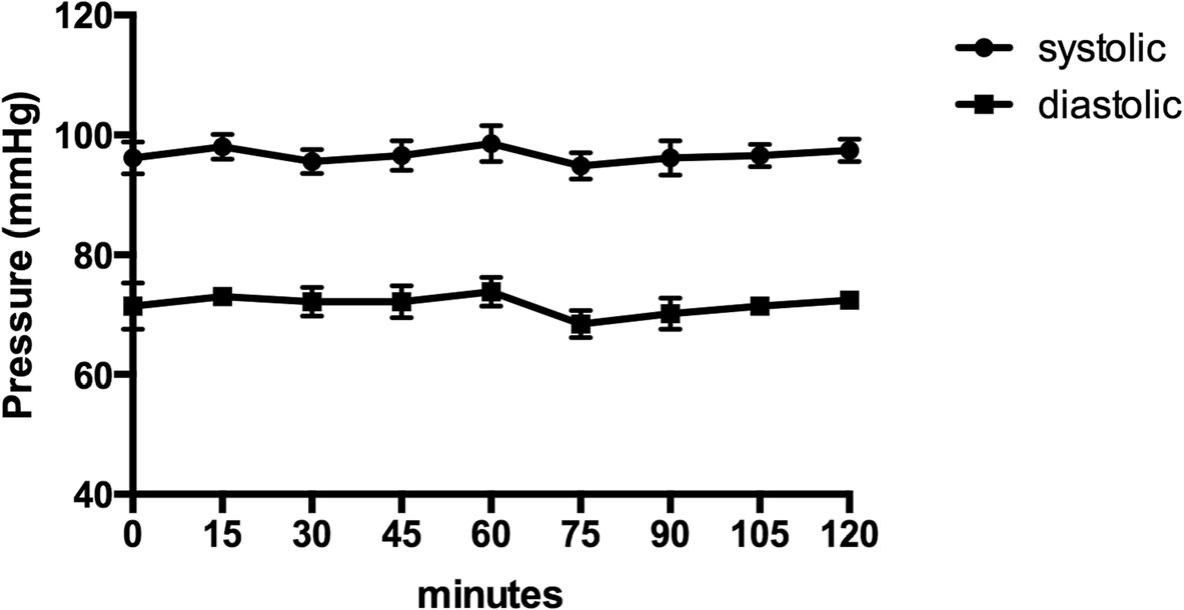
Systolic and diastolic arterial blood pressure during desflurane anesthesia. Arterial blood pressures were stable throughout anesthesia. Data are presented as mean ± SEM (*n* = 5)

Throughout desflurane exposure, HR, SaO_2_, and the temperatures were within the normal range (data not shown) and arterial blood pressures were stable (Fig. 2). The values of pH, PaCO_2_, PaO_2_, and HCO_3_^−^ were 7.38 ± 0.02, 42.0 ± 2.8, 185.0 ± 11.1, and 24.0 ± 0.5 (mean ± SEM), respectively after 2 h of anesthesia.

### IA latencies on day 1 were significantly declined with 1.2 MAC desflurane, but not with 0.7 MAC

**Fig. 3:**
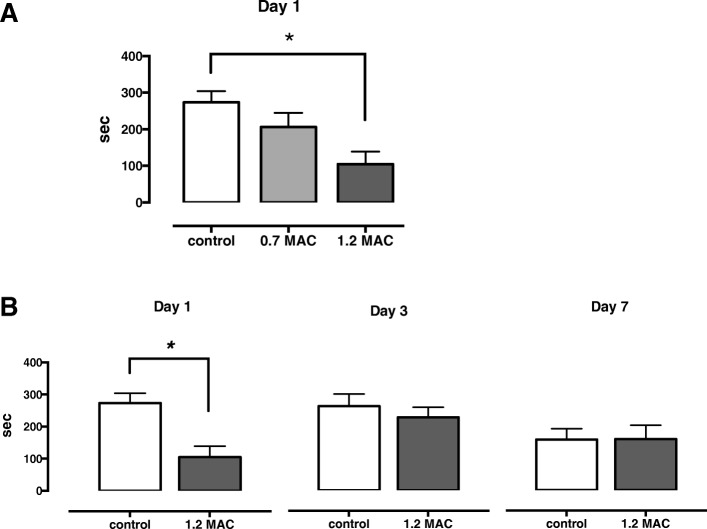
IA latencies. (**a**) IA latencies on day 1 were significantly declined in the 1.2 MAC desflurane (*n* = 17, 104 ± 34 s) (post hoc Tukey’s, _*_P = 0.004), but not in the 0.7 MAC desflurane group (*n* = 17, 206 ± 39 s) (post hoc Tukey’s, *P* = 0.362) compared with the control (*n* = 17, 273 ± 31 s). (**b**) IA latencies were not declined on day 3 (control [*n* = 16], 264 ± 38 s; desflurane [*n* = 16], 229 ± 32 s) and day 7 (control [*n* = 15], 159 ± 35 s; desflurane [*n* = 15], 160 ± 44 s) (two-way ANOVA, group, *F* [1,90] = 5.426, *P* = 0.022; day, *F* [2,90] = 0.052, *P* = 0.417; group x day, *F* [1,90] = 3.263, *P* = 0.043, post hoc Sidak’s multiple comparison test, day 3, control vs. desflurane, *P* = 0.861; day 7, control vs. desflurane, *P* > 0.999). Each rat was tested only once. The same data are used for day 1 in (**a**) and (**b**). Data are presented as mean ± SEM

The latency to enter the dark box on day 1 after desflurane exposure significantly declined in the 1.2 MAC desflurane group (*n* = 17, 105 ± 34 s), but not in the 0.7 MAC desflurane group (*n* = 17, 206 ± 39 s), compared with the control (*n* = 17, 273 ± 31 s; one-way ANOVA, *F* [2,48] = 5.974, *P* = 0.005, post hoc Tukey’s multiple comparison test, mean difference [95% confidence interval], control vs. 1.2 MAC desflurane, 168 [49.9 to 287], *P* = 0.004; 1.2 MAC desflurane vs. 0.7 MAC desflurane, − 101 [− 220 to 17.7], *P* = 0.110; control vs. 0.7 MAC desflurane, 67.5 [− 51.2 to 186], *P* = 0.362; Fig. 3a).

### IA latencies were not declined on days 3 or 7 with 1.2 MAC desflurane

To investigate whether the impairment of learning after exposure to 1.2 MAC desflurane is a long lasting change or a temporary reversible change, we performed IA test on days 3 and 7 after exposure to 1.2 MAC desflurane. The latencies of the desflurane group were not significantly different from those of the controls on days 3 and 7 (day 3, control [*n* = 16], 264 ± 38 s; desflurane [*n* = 16], 229 ± 32 s; day 7, control [*n* = 15], 159 ± 35 s; desflurane [*n* = 15], 160 ± 44 s, two-way ANOVA, group, *F* [1,90] = 5.426, *P* = 0.022; day, *F* [2,90] = 0.052, *P* = 0.417; group x day, *F* [1,90] = 3.263, *P* = 0.043, post hoc Sidak’s multiple comparison test, day 3, control vs. desflurane, *P* = 0.861; day 7, control vs. desflurane, *P* > 0.999) (Fig. 3b).

Collectively, these results showed that exposure to 1.2 MAC concentration of desflurane caused short-term reversible impairments in contextual learning.

### Hippocampal LTP was significantly suppressed on day 1 of desflurane exposure

**Fig. 4:**
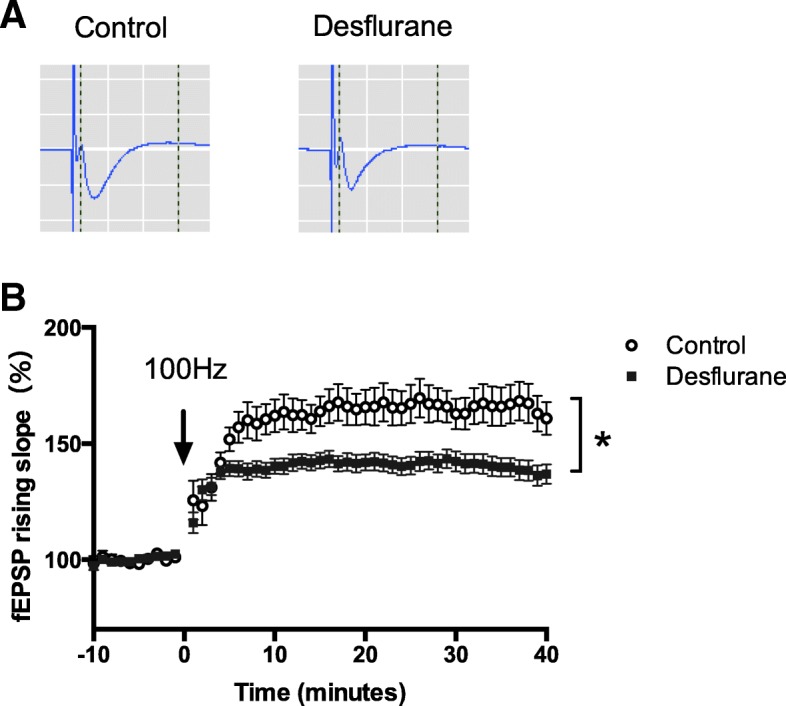
Hippocampal LTP was significantly suppressed on day 1 of desflurane exposure. (**a**) Representative fEPSP traces following the induction of stable LTP. (B) Summary of the fEPSP slopes after applying high-frequency stimulation (100 Hz, 1 s). LTP, induced by the stimulation was significantly suppressed after desflurane exposure (control [*n* = 5], 165 ± 8%; desflurane [*n* = 7], 138 ± 4%; _*_P = 0.006)

Because contextual learning was impaired on day 1 of desflurane exposure, we assessed the LTP of hippocampal CA1 to confirm the changes in synaptic plasticity. Hippocampal LTP on day 1 was significantly suppressed in the desflurane group (*n* = 7, 138 ± 4%) compared with the control group (*n* = 5, 165 ± 8%; *P* = 0.006; Fig. 4).

### Synaptic GluR1 did not increase by IA-training on day 1 of desflurane exposure

IA training is known to induce LTP in the hippocampus by delivering AMPAR to synapse [22, 23]. Because LTP was suppressed with desflurane exposure, we investigated the expression levels of the AMPAR GluR1 subunit on day 1 in both IA-trained and untrained groups. One rat in the IA-trained desflurane group stayed in the light box until the cutoff time of 360 s during the training phase and was not shocked. Thus, this animal was excluded.

**Fig. 5:**
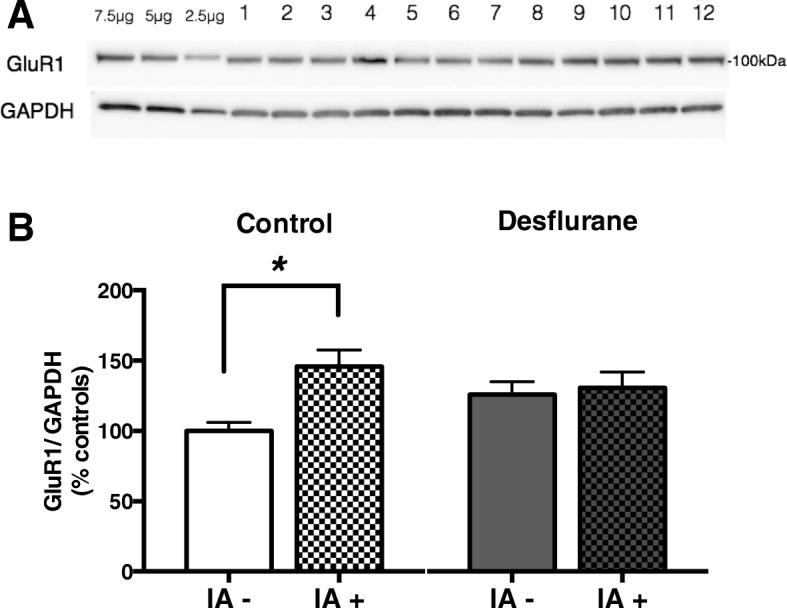
GluR1 was not elevated with IA-training on day 1 of desflurane exposure. (**a**) Representative immunoblot of GluR1 in synaptoneurosomes. The first three bands were used to create calibration curve. Lanes 1, 5, 9: IA-trained control; lanes 2, 6, 10: IA-trained desflurane; lanes 3, 7, 11: untrained control; lanes 4, 8, 12: untrained desflurane. (**b**) The amount of GluR1 in synaptoneurosomes was significantly elevated with IA-training in the control (145.9 ± 11.6% of untrained control; post hoc Sidak’s test, _*_P = 0.003), but not in the desflurane (103.8 ± 8.9% of untrained desflurane; post hoc Sidak’s test, *P* = 0.927), group

There was no difference between the untrained control and untrained desflurane group (125.9 ± 9.2% of untrained control) (group x IA, *F* [1, 28] = 4.490, *P* = 0.041; group, *F* [1, 28] = 0.305, *P* = 0.585; IA, *F* [1, 28] = 6.851, *P* = 0.013; post hoc Sidak’s multiple comparison test, untrained control vs. IA-trained control, *P* = 0.010; untrained desflurane vs. IA-trained desflurane, *P* = 1.000; untrained control vs. untrained desflurane, *P* = 0.326; Fig. 5). After the IA training, the GluR1 protein level was significantly elevated in the control group ([*n* = 10], 145.9 ± 11.6% of untrained control group [*n* = 10]). In contrast, the GluR1 protein level was not elevated with IA training in the desflurane group ([*n* = 9], 103.8 ± 8.9% of untrained desflurane group [*n* = 10]). These results indicate that desflurane does not alter baseline synaptic GluR1 expression, but suppresses the delivery of GluR1 to hippocampal synaptoneurosomes caused by IA training.

## Discussion

The present study has showed that exposure to 1.2 MAC desflurane for 2 h caused impairment of contextual learning on day 1, but not on day 3 or 7. The concentration of desflurane appears important because 0.7 MAC desflurane has no effects on day 1. The impairment in learning on day 1 is accompanied by suppression of hippocampal LTP. Moreover, desflurane inhibits the delivery of AMPAR to synapses produced by IA training without altering baseline synaptic GluR1 expression.

These results are similar to those of isoflurane we demonstrated in our previous study [15]. However the distinct difference is that the learning impairment by desflurane occured and dissipated much earlier than that of isoflurane (day 1 vs. day 7 after exposure). The mechanisms underlying this difference remain unclear, but are unlikely to be explained by the differences in the blood/gas partition coefficients and the resulting differences in the pharmacokinetics of the two anesthetics, because the observed learning impairments occurred long after the anesthetics disappeared from the central nervous system. Several studies have reported that in contrast to isoflurane [20, 21], desflurane does not induce mitochondrial damage [16], apoptosis [17, 18], caspase activation, or amyloid β generation [19] in cells, neurons, or tissues, which suggests that desflurane is less likely than isoflurane to have lasting effects on the central nervous system.

We believe that our finding of desflurane causing learning deficits on day 1 in young adults is relevant, because quick and complete recovery and earlier discharge are required in the ambulatory surgery setting, presumably more so in the working age patients than in older patients. Nearly half of ambulatory surgery patients are reported to resume normal activities on postoperative day 1 [28, 29]. Our results imply that patients may have the risk of cognitive decline on the hours following surgery.

Several clinical studies have compared desflurane with sevoflurane [6, 7, 9], isoflurane [4], or propofol [5, 8] on cognitive function after general anesthesia. Some studies have reported the superiority of desflurane, while other studies have reported no differences, regardless of the time when the neurocognitive tests were performed, as the postoperative tests were performed within 24 h in some studies [6–9], and after 24 h in others [4–6, 8]. Accordingly, the effects of desflurane on cognitive dysfunction in clinical settings remain unclear. The concentration of desflurane may have meaning, as learning was impaired dose-dependently, which is consistent with the previous animal studies [14, 15, 29, 30], and deep anesthesia has been shown to increase postoperative delirium [30, 31], POCD [31, 32], and 1-year mortality [32, 33] clinically.

Our immunoblot analysis has showed that desflurane prevented the increase in synaptoneurosomal GluR1 induced by IA training. This suggests that prior exposure to desflurane interferes with AMPAR trafficking [22], which is known to be essential for the establishment of hippocampal learning. We previously reported that exposure to isoflurane increased GluR1 significantly in the untrained group by inhibiting ubiquitination, a main degradation pathway of GluR1 [15]. By contrast, exposure to desflurane showed no significant differences in GluR1 between the untrained control and desflurane groups. The mechanisms by which desflurane prevents the delivery of AMPAR to synapses are unclear, and as the molecular processes involved in AMPAR trafficking are exceedingly complex [33–35], further studies are needed to elucidate the underlying mechanisms by which desflurane prevents the delivery of AMPAR to synapses.

There are limitations to our study. First, we employed only one behavioral test of contextual learning (the IA test), and therefore, did not evaluate other aspects of cognitive function. Second, no group of rats underwent surgery, because we wished to examine the effects of desflurane alone. As inflammation due to surgery is known to cause learning and memory impairment [28, 35], learning deficits caused by desflurane may have been exacerbated and/or prolonged if surgery was superimposed. Third, we examined the AMPAR in the current study because it is known to be critical in learning and memory. However, our results do not preclude the possible role of other receptors such as γ-aminobutyric acid [36] and *N*-methyl-D-aspartic acid receptors [37], which are known to be the important targets of inhalational anesthetics.

## Conclusions

Exposure to 1.2 MAC desflurane for 2 h caused short-term reversible impairment of learning and memory in young adult rats accompanied by suppression of LTP and the delivery of GluR1 to hippocampal synaptoneurosomes. To fully evaluate the short-term effects of inhalational anesthetics on learning and memory, further studies are required in other inhalational anesthetics (e.g. sevoflurane or xenon).

## Data Availability

The data of the current study are available from the corresponding author on reasonable request.

## Abbreviations

ACSF: artificial cerebrospinal fluid
AMPAR: α-amino-3-hydroxy-5-methyl-4-isoxazolepropionic acid receptor
ANOVA: analysis of variance
fEPSPs: extracellular field excitatory postsynaptic potentials
HR: heart rate
IA: inhibitory avoidance
LTP: long-term potentiation
MAC: minimum alveolar concentration
POCD: postoperative cognitive dysfunction
POD: postoperative delirium
SaO_2_: oxygen saturation
SEM: standard error of mean
